# Enhancing Efficiency, Stability, and Cycle Life of Lithium Metal Electrodeposition in Dry Solid-State Polymer Electrolytes

**DOI:** 10.1021/acsami.4c15287

**Published:** 2024-11-20

**Authors:** Idan Bar-lev, Keren Shwartsman, Vivek Kumar Singh, Netta Bruchiel-Spanier, Emily Ryan, Netanel Shpigel, Daniel Sharon

**Affiliations:** † Institute of Chemistry, The Hebrew University of Jerusalem, Jerusalem 9190401, Israel; ‡ Department of Mechanical Engineering; Division of Materials Science and Engineering; Institute for Global Sustainability, Boston University, Boston, Massachusetts 02215, United States; § Department of Chemical Sciences, Ariel University, Ariel 40700, Israel

**Keywords:** Energy storage, Li-metal batteries, solid-state electrolytes, polymer electrolytes, electrodeposition, Coulombic efficiency

## Abstract

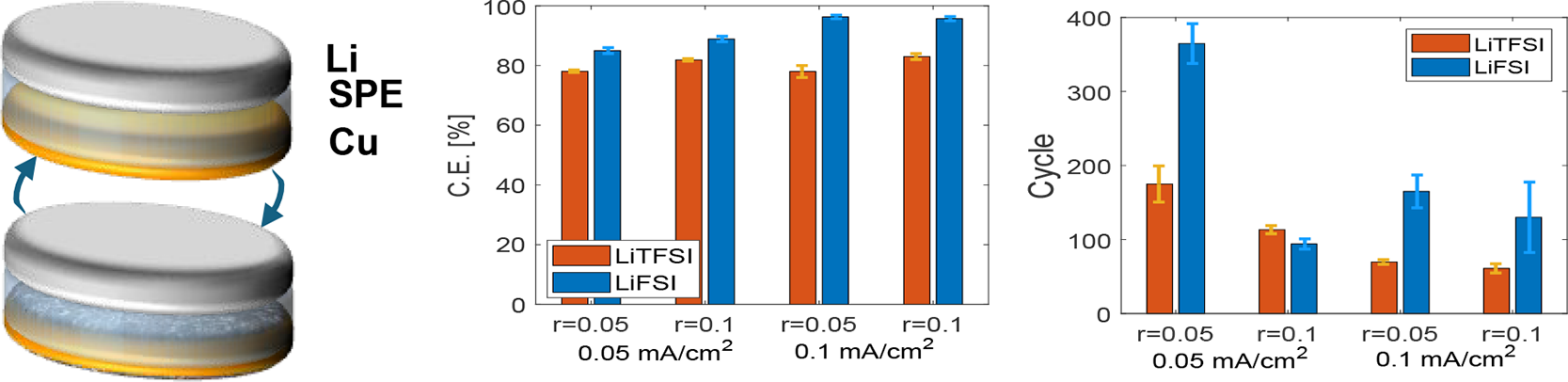

Dry solid polymer electrolytes (SPEs), particularly those based on poly(ethylene oxide) (PEO), hold significant potential for advancing solid-state Li-metal battery (LMB) technology. Despite extensive research over the years, a comprehensive evaluation of Coulombic efficiency (CE), deposit stability, and cycle life for reversible Li metal electrodeposition in SPE-based cells is still lacking. In this study, we systematically assess the effect of cycling conditions on the CE of Li|SPE|Cu half cells and provide a thorough examination of different electrolyte chemistries, highlighting and explaining their performance across various parameters. While the efficiency of the PEO-based SPEs still falls short of the efficiency benchmark set by liquid and gel electrolytes, we demonstrated >95% CE with Lithium bis(fluorosulfonyl)imide (LiFSI)-based SPEs, surpassing previous reports for dry SPEs in a Li|SPE|Cu cells, this result marks a significant breakthrough. Furthermore, our findings highlight the critical impact of the Li-SPE interphase on these performance metrics. The LiFSI-based SPE forms a Li-rich, high-conductivity interphase, which not only enhances efficiency but also improves cycle life and Li deposit stability. These results underscore the importance of selecting the right polymer electrolyte chemistry and concentration to enhance SPE performance.

## 1 Introduction

Liquid-based Li-ion batteries have established a robust foundation for current energy storage solutions, but their limitations in energy density and safety concerns have driven the search for innovative alternatives. Solid-state Li metal batteries (LMBs) emerge as a pivotal solution in this regard, offering the potential to significantly enhance both safety and energy density.^1^ By facilitating the use of high energy density Li metal anodes, solid-state electrolytes could be the key to advancing beyond current energy storage capabilities. This shift not only aims to mitigate the inherent risks associated with liquid electrolytes, such as flammability but also could potentially address the challenges of dendritic growth associated with Li metal, thus paving the way for the next generation of high-energy-density batteries.^2,3^

Dry solid polymer electrolytes (SPEs), particularly those based on poly(ethylene oxide) (PEO), were recognized many years ago as a potential material system for constructing solid-state LMBs.^4^ However, despite the long-standing research into SPEs for LMBs, comprehensive insights into their efficiency and cycle life, particularly in the context of reversible Li electrodeposition, remain notably limited. SPE studies almost exclusively investigate full-cell (Li|SPE|Cathode) or symmetric cell (Li|SPE|Li) configurations with thick Li metal foils. Yet, the presence of electrodes with reservoirs of metallic lithium presents significant challenges in accurately evaluating reversible electrodeposition efficiency, stability, and the overall cycle life. The complete stripping and deposition of Li onto a Cu substrate can enable our investigation to focus on many of the processes that dominate cell performance such as solid electrolyte interphase (SEI) formation and Li nucleation in SPE half cells.

Recent insights into this field have been highlighted by half-cell studies (Li|SPE|Cu), such as those conducted by Zhang et al., who reported a Coulombic efficiency (CE) of just 30% in cells containing dry SPEs, and Bertoli et al., who documented efficiencies ranging from 40 to 70% in Li-based SPEs formulated with zinc salt additives.^5,6^ Although these studies provide valuable data, they also expose a substantial research gap in understanding and enhancing the crucial performance metrics for SPEs’ practical application in LMBs. Moreover, it is critical to establish a clear correlation between the cycle life and efficiency of reversible electrodeposition and the Li-SPE interphase composition to advance the development of more stable and efficient SPE systems. Notably, the performance disparity becomes more pronounced when contrasting dry SPEs with cells based on liquid and gel electrolytes, where efficiencies have surpassed the >99.9% benchmark necessary for rechargeable battery technologies.^7^

The literature on liquid electrolyte solutions highlights the paramount importance of the SEI formed during cycling and its impact on CE.^8−10^ An unstable interphase can lead to decreased CE and a reduced cycle life, whereas an optimal SEI composition can significantly improve these performance metrics. Yet most of the conducted studies are related to liquid or composite electrolytes, while systematic research on the efficiency and lifetimes of dry SPEs is lacking. Moreover, the interplay between the SEI stability and electrolyte degradation processes further complicates the performance dynamics of LMBs.^11−13^ Corrosion can severely degrade the interphase quality, exacerbating capacity loss and diminishing cycle life. Therefore, alongside efficiency and cycle life evaluations, an analysis of the electrode-SPE interphase, including corrosion effects, is imperative for enhancing the SPE performance.

In this context, our study seeks to be the first to systematically evaluate the performance of Li|SPE|Cu half-cells, focusing on the impacts of fabrication methods, electrolyte compositions, and cycling conditions on CE, deposit stability, and cycle lifetime, establishing clear performance benchmarks. By exploring the use of lithium bis(trifluoromethanesulfonyl)imide (LiTFSI) and lithium bis(fluorosulfonyl)imide (LiFSI) within PEO-based SPEs, this study aims to elucidate the physicochemical factors that influence the efficiency and stability of lithium metal electrodeposition.^14,15^ Addressing these factors is crucial for advancing the design and functionality of next-generation solid-state LMBs, pushing the boundaries of energy density, safety, and durability in energy storage technologies.

## 2 Results and Discussion

The two parameters commonly employed to describe the reversible electrodeposition performance of Li metal are Coulombic efficiency (CE) and the cycle life preceding cell failure.^16^ CE serves as a crucial metric in rechargeable batteries, quantifying the efficiency of reversible electrochemical reactions or processes. Specifically, in the context of Li-metal anodes, CE reflects the efficiency of Li deposition and stripping processes over successive cycles.^17^ Although various protocols exist for determining CE, the most straightforward method involves repeated deposition and stripping of Li metal on a foreign substrate, such as copper, which is the most common current collector for Li-anode materials. The CE is then calculated as the ratio of the charge obtained during stripping to that during deposition, expressed as (*Q*_stripping_/*Q*_deposition_) × 100%. Notably, for a durable rechargeable LMBs, the CE need to exceed 99.9%.^7^ Monitoring and enhancing CE are vital for advancing LMBs technologies and ensuring dependable, efficient energy storage.^7,17,18^ The cycle life of reversible electrodeposition represents the average number of cycles before cell failure, which occurs upon experiencing either a soft or hard short-circuit.^19,20^ Given the stochastic nature of dendritic growth, precisely predicting the cycle life is challenging. Therefore, using a substantial number of cells is necessary to obtain a reliable average value. Our methodology, which averages results from numerous identical cells, enhances the reliability of our findings.

LiTFSI is notably the most frequently utilized electrolyte paired with poly(ethylene oxide) (PEO) in dry solid polymer electrolytes (SPEs). High ionic dissociation endows LiTFSI with significantly greater ionic conductivity in PEO-based polymers compared to other salts traditionally employed in liquid electrolytes.^15^ Recent investigations into liquid electrolyte systems have revealed that LiFSI may afford superior CE during Li metal electrodeposition, outperforming LiTFSI-based electrolyte solutions.^21^ Although the chemistry of both anions is quite similar, it is been suggested that the presence of different fluorine functional groups can significantly alter the chemical characteristics of the SEI.^22,23^ Moreover, SPEs incorporating LiFSI have been reported to enhance the performance of solid-state LMBs.^21,24^ Regarding the salt molar concentration of SPEs, the ratio ([Li]/[EO]) of Li to ethylene oxide (EO) units typically stands at [Li]/[EO] = 0.05 or [Li]/[EO] = 0.1, which may result in higher mechanical robustness or increased ionic conductivity, respectively.^25,26^ The specific impacts of these salts and their concentrations on the efficiency and cycle life of reversible lithium electrodeposition in SPE-based cells remain underexplored, underscoring the necessity for comprehensive evaluations and analysis.

**Figure 1:**
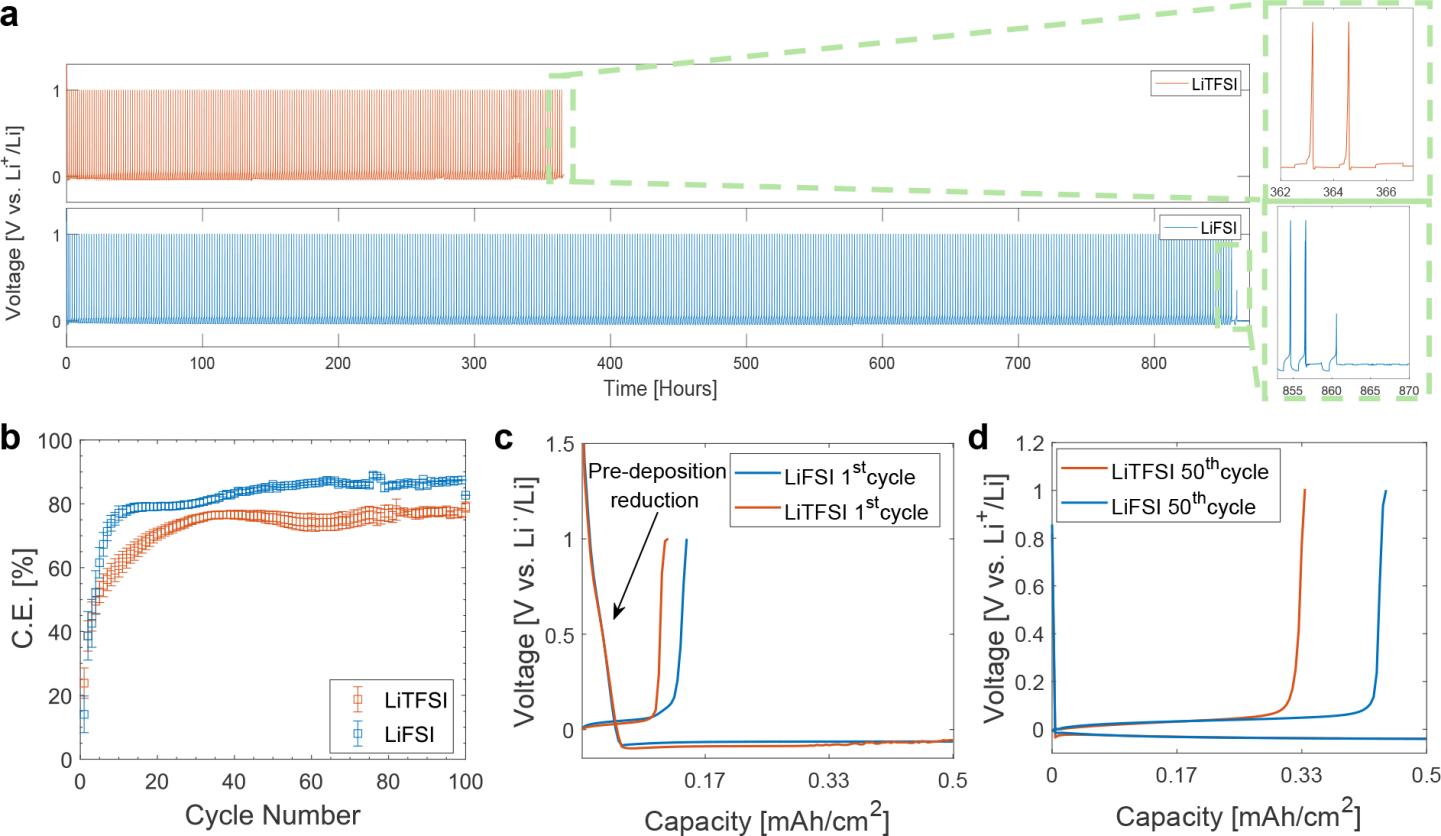
(a) Cycling voltage profiles of SPE-based cells with LiTFSI or LiFSI concentration ratio of [Li]/[EO] = 0.05 at current density of 0.05 mA/cm^2^ and 60 °C, short circuit presented in the inset for each type of cell. (b) CE over the first 100 cycles. Voltage profiles of the (c) initial and (d) fiftieth cycles.

We first focused on the reversible electrodeposition behavior of Li|SPE|Cu cells incorporating 100 μm dry SPE membranes with LiTFSI or LiFSI ([Li]/[EO] = 0.05) under a current density of 0.05 mA/cm^2^ and a capacity of 0.05 mAh/cm^2^. All cells were cycled at 60 °C, above the melting point of PEO. The cycling profiles, illustrated in Figure 1a, initially revealed similar deposition and stripping curves for both systems. Notably, the cells containing LiTFSI-based SPEs demonstrated earlier cell failure compared with their LiFSI counterparts (Figure 1a). Under these conditions, LiFSI-based cells exhibited an extended cycle life of 350 ± 30 cycles, while LiTFSI-based cells demonstrated a shorter lifespan of only 180 ± 20 cycles before failure. Looking at the calculated average CE from these cycling profiles, we observe that cells employing both types of membranes experienced a gradual increase in CE, reaching a stable plateau, as shown in Figure 1b. Specifically, stabilization for LiFSI-based cells occurred at 85 ± 1% CE after approximately 15 cycles, whereas LiTFSI-based cells required closer to 30 cycles to achieve a lower CE of 78.1 ± 0.4%. The average CE was calculated over the last 50 cycles before short-circuit. While differences in ionic conductivity often contribute to variations in performance across SPE-based batteries, we observed comparable conductivities for both salts in this study (see Figure S2). Additionally, the measured transference numbers were similar, with the LiTFSI-based membrane at *t*_+_≈ 0.147 and the LiFSI-based membrane slightly lower at *t*_+_≈ 0.144 (see Figure S3). These results indicate that the primary differences in performance between the two cell types are likely due to variations at the SPE-Li metal interphase rather than differences in the bulk properties of the SPEs.

The voltage profile during the first electrodeposition process, shown in Figure 1c, noticeably differs from the one following 50 cycles after the CE has stabilized, as seen in Figure 1d. During the first electrodeposition process, both types of cells exhibit a notable electrochemical response at voltages earlier than the thermodynamic potential of Li electrodeposition. Notably, the initial reduction for both SPEs is marked by a decrease in incline of the potential curve near ∼0.8 V vs Li. It is proposed that PEO-based polymers experience partial reduction, along with the electrolyte anion, accounting for the initial reduction process that precedes the stable voltage plateau characteristic of Li electrodeposition.^13^ While the initial voltage reduction profiles are similar for both salts, subsequent analysis will reveal that the reduction products and their concentrations differ between the two types of salts. After 5 cycles, the reduction reactions above 0 V vs Li become less evident for both salts, resulting in a sharp decrease in voltage before stabilizing at the overpotential required for lithium deposition.

**Figure 2:**
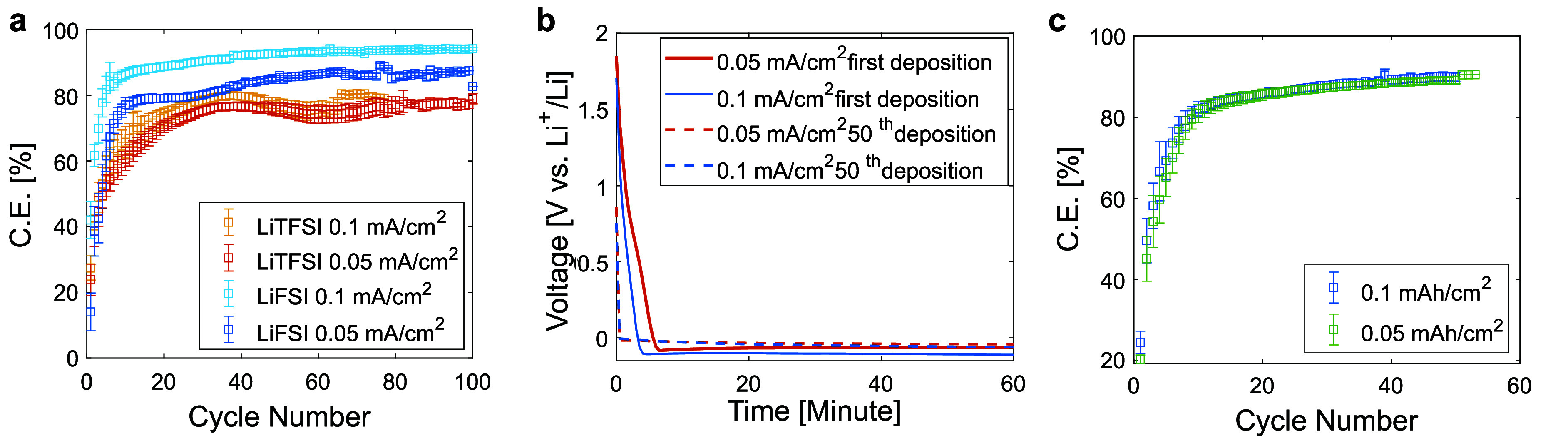
(a) CE as a function of cycle number for SPE-based cells at a concentration ratio of [Li]/[EO] = 0.05 under various current densities at 60 °C. (b) Corresponding voltage profiles of the cells based on LiFSI-SPEs at different current densities. (c) CE as a function of cycle number for cells with [Li]/[EO] = 0.05, operated at a current density of 0.1 mA/cm^2^ and varying capacities.

The electrodeposition behavior of Li is profoundly influenced by the rate of deposition dictated by the applied current density. At higher current densities, the nucleation and growth mechanisms of lithium deposits typically result in disordered and irregular deposit layers.^27^ Such nonuniform morphologies, including dendritic structures, can significantly reduce CE by breaking off and creating “dead Li”, this can also lead to premature cell failure. It is important to recognize that most of these observations and trends come from studies that use liquid electrolyte solutions. Due to their relatively lower ionic conductivity, dry polymer electrolytes typically underperform at higher current densities compared to their liquid counterparts.^28^ Our findings reveal that SPE-based cells subjected to current densities exceeding 0.15 mA/cm^2^ encountered rapid failure within a few cycles, underscoring their limitations in providing dependable insights (Figure S4). Consequently, we increase the current density to 0.1 mA/cm^2^ for cells with both LiFSI and LiTFSI-based SPEs ([Li]/[EO] = 0.05), as depicted in Figure 2. As expected, this adjustment resulted in a reduced cycle lifespan for SPE cells, with LiFSI-based cells lasting for 140 ± 10 cycles and LiTFSI-based cells lasting for 75 ± 3 cycles.

Increasing the current from 0.05 to 0.1 mA/cm^2^ resulted in no significant change in CE for cells with LiTFSI-based SPEs (Figure 2a). However, in the case of LiFSI-based SPEs, an increase in current notably improved the CE from 85 ± 1% to 91.3 ± 0.2% after cell stabilization. Higher current induces a more rapid voltage decline during the first electrodeposition compared with the initial profile observed in cells cycled at a lower current density of 0.05 mA/cm^2^ (Figure 2b). This quicker voltage decline suggests kinetic control over some of the reduction reactions occurring before lithium metal electrodeposition. These processes have been linked to the reduction of the polymer electrolyte and the subsequent potential passivation of the electrode, impacting to composition of the SEI.^29^ A decrease in early reduction reactions was also observed in LiTFSI-based reactions (Figure S5), although it did not result in enhanced efficiency. This suggests that the impact on CE is not primarily driven by the extent of side reactions during the initial electrodeposition process, but it more directly linked to the chemical nature of the SEI formed.

The improvement in CE at higher current density observed in LiFSI-based cells may also be related to the higher amount of deposited Li.^7^ To ascertain that the increase in CE is not attributable to increased capacity, we cycled cells at 0.1 mA/cm^2^ at a capacity of 0.05 mAh/cm^2^, equal to that of the cells cycled at the lower current density of 0.05 mA/cm^2^. As depicted in Figure 2c, changes in Li capacity have a negligible effect on CE. This observation reinforces the idea that the key to enhancing CE in LiFSI-based cells is caused by the increase of current density rather than by the increase in the capacity, affirming the critical role of current density in the electrochemical performance of the cell.

**Figure 3:**
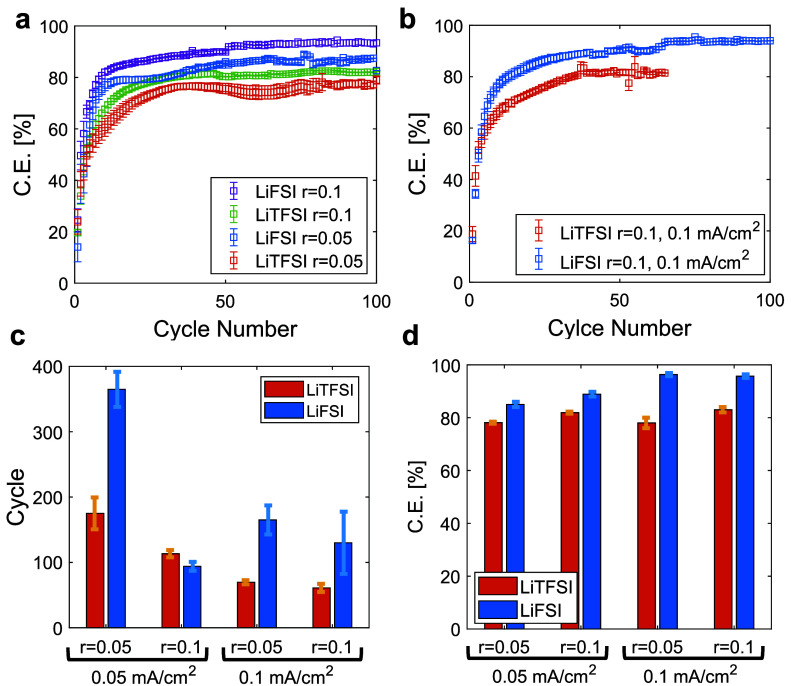
(a) CE during cycling of SPE-based Li metal cells with different salt concentration at current density of 0.05 mA/cm^2^ and 60 °C. (b) CE during cycling of SPE-based Li metal cells at a salt concentration of r = 0.1 and current density of 0.1 mA/cm^2^ (c) Average cycle lifetime and (d) average CE of Li-metal SPE-based cells. The average CE was determined from the last 50 cycles.

Up to this point, our investigation has primarily focused on a concentration ratio of [Li]/[EO] = 0.05 [Li]/[EO]. While increasing the salt concentration to [Li]/[EO] = 0.1 increases Li ion availability for reaction, it may compromise the mechanical integrity of the membrane.^25,26,30−32^ Although the impacts of concentration on ionic conductivity and mechanical properties are acknowledged, their influence on the cycle life and efficiency of reversible electrodeposition in dry SPEs-based cells remains less understood. To address this gap, we have extended our investigation to higher concentrations, specifically examining the [Li]/[EO] = 0.1 ratio in both LiTFSI and LiFSI-based SPEs. The shift to a higher concentration ratio of [Li]/[EO] = 0.1 resulted in significant improvements in CE for both types of SPEs. CE for LiTFSI-based SPEs increased from 78.1 ± 0.4% to 81.9 ± 0.4%, while LiFSI-based SPEs increased from 85 ± 1% to 88.9 ± 0.9% at current density of 0.05 mA/cm^2^ (Figure 3a). The improvements in CE observed with an increased salt concentration can be linked to various factors. One factor can be the enhanced availability of lithium ions at the electrode interface. This enhancement can facilitate a more stable electrodeposition, thereby improving the process reversibility.

While the increased salt concentration can enhance efficiency, it may also lead to a decrease in cell cycle life. This effect is particularly evident in SPE-based cells with LiTFSI salt, as shown in Figure 3c. Furthermore, increasing the salt concentration beyond *r* > 0.1 did not result in further improvements but instead diminished both the CE and cycle life of the Li electrodeposition process, as shown in Figure S6. Possible explanations for the decline in performance include the deterioration of the SPE’s mechanical integrity and the decreased ionic conductivity at higher salt concentrations^33^ These findings highlight the challenge of improving CE with highly concentrated SPEs, as it may lead to reduced cycle life. Therefore, it is vital to develop strategies that allow for higher salt concentrations without negatively affecting longevity.

To summarize the findings, data have been compiled to showcase the trends in the cycle life (Figure 3c) and average CE (Figure 3d) across various sample types. It was found that LiFSI-based SPE cells exhibit higher CE compared to LiTFSI-based cells across the board, with CE increasing alongside current density and concentration. However, lower current densities have a tendency for longer cycle life. An increase in salt concentration led to higher average CE, but inversely affected cycle life, presenting a challenge in enhancing both metrics concurrently. A key achievement is attaining a CE of 96.3 ± 0.6% with PEO-LiFSI at a concentration ratio of [Li]/[EO] = 0.1 and a current density of 0.1 mA/cm^2^, setting a new benchmark for dry homopolymer PEO-based solid electrolytes without additives (Figure 3b). While the CE has not yet reached the >99.9% efficiency required for practical rechargeable battery applications, this result represents a valuable step forward.^7^ Progressing toward this target demands an in-depth understanding of the efficiency-enhancing mechanisms, particularly the differences observed between SPEs formulated with LiFSI and LiTFSI salts.

**Figure 4:**
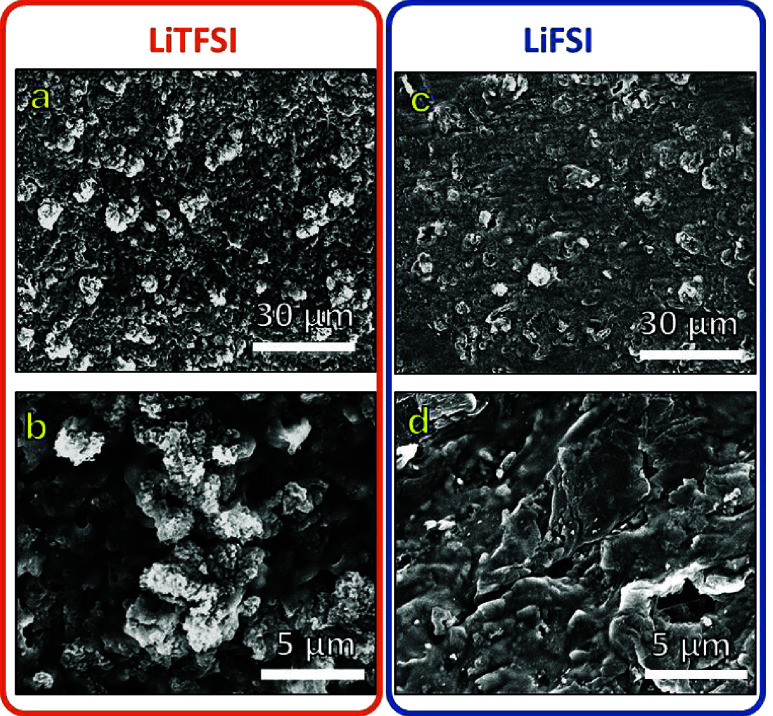
SEM images of Li deposits on the SPE surface that was in direct contact with the Cu substrate. (a-b) LiTFSI-SPE after Li deposition at 0.05 mA/cm^2^. (c-d) LiFSI-SPE after Li deposition at 0.05 mA/cm^2^.

To further explore the differences between the two salts, we imaged the lithium morphology from SPE-based cells after deposition by using scanning electron microscopy (SEM). The SEM images presented in Figure 4 showcase the deposited lithium layer on the SPE surface that was in direct contact with the Cu substrate. Notably, the SPE based on LiFSI exhibits a denser and smoother deposited lithium layer (Figures 4a and 4b) compared to that of LiTFSI (Figures 4c and 4d). We note that while SEM imaging provides valuable insights, it poses challenges as the polymer electrolyte cannot be separated from the Cu substrate without disturbing the adhered Li metal, potentially affecting the observed morphology. Despite this limitation, the images provide critical clues about the deposition behavior. The increased deposit density in LiFSI-based SPEs likely contributes to a reduction in ’dead Li’ formation, thereby enhancing CE.^34^ Additionally, formation of a more compact deposit layer helps to suppress uneven Li growth, contributing to extended cycle life.^35^

**Figure 5:**
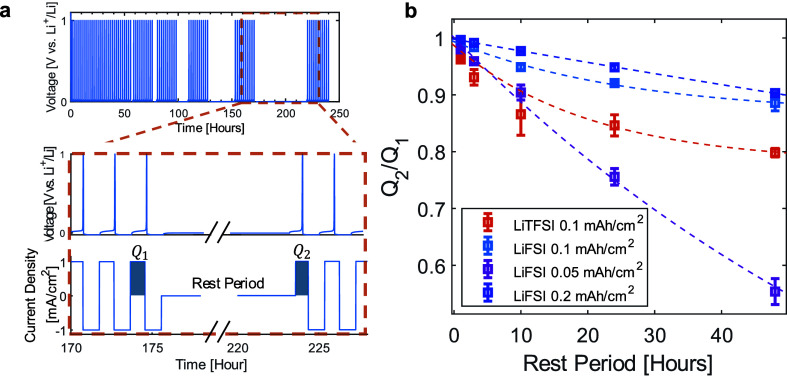
(a) Representative protocol for the self-discharge evaluation of deposited Li metal in SPE-based cells. (b) Capacity retention (*Q*_2_/*Q*_1_) as a function of rest period for deposited Li metal in different SPE-based cells ([Li]/[EO] = 0.05) cycled at 60 °C.

To evaluate the stability of electrodeposited Li on the Cu substrate across each SPE system, we undertook detailed corrosion measurements. Corrosion of the plated lithium occurs due to its reaction with the SPEs, leading to capacity loss during storage, a phenomenon often referred to as self-discharge. As illustrated in Figure 5a, the self-discharge protocol begins with 30 stabilization cycles, after which Li is deposited onto the Cu substrate. The cell is then rested for various time intervals, after which the remaining Li is stripped (*Q*_2_) and compared to the stripping process after stabilization (*Q*_1_) to calculate the capacity loss during storage. Following this, the cell undergoes 10 additional stabilization cycles before introducing a longer rest period.

As shown in Figure 5b, the capacity loss increases with longer rest durations in both SPE-based cells. Notably, the LiFSI-based system exhibits significantly better stability, showing less capacity loss than the LiTFSI-based system. This difference becomes highly visible over extended storage periods, with LiTFSI-based SPE cells showing a 20.2 ± 0.7% loss in capacity after 48 h, while LiFSI-based SPE cells experience only an 11 ± 1% loss. As shown by the dashed fitting curves, the charge loss over storage time follows a first-order kinetics model, as proposed by Yazami et al.^36^ where capacity decay is described by
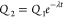
Here, *Q*_2_ represents the retained capacity after the self-discharge period, and *Q*_1_ is the initial capacity obtained after stabilization and before self-discharge. The decay constants, λ, for the LiTFSI and LiFSI systems were found to be (4.2 ± 0.7) × 10^–3^ h^–1^ and (3.0 ± 0.5) × 10^–3^ h^–1^, respectively. For LiTFSI, this corresponds to a cell half-life of approximately 165 h, while for LiFSI, it results in a longer half-life of 231 h. This difference highlights the impact of the resting time on the integrity of the deposited Li layer and the stability of the SPE-Li interface generated by each salt. The superior resilience of the LiFSI-based system suggests its enhanced ability to form a more robust and protective SEI layer, which helps prevent further degradation of both the SPE and the lithium deposit.^11^ This, in turn, we propose, contributes to the improved efficiency of LiFSI-based SPEs.

The loss of the plated Li metal can occur through both galvanic and chemical corrosion processes. Galvanic corrosion requires contact between the SPE, plated Li, and the exposed Cu substrate, leading to a redox reaction in which the Cu substrate reduces the SPE, thereby accelerating the oxidation of the Li metal.^12,13,37−39^ Chemical corrosion, on the other hand, involves the direct oxidation of Li by the SPE. To differentiate between the effects of galvanic and chemical corrosion, we assessed the role of the capacity in self-discharge. Higher capacities, resulting from greater amounts of Li deposition, are thought to minimize exposure of the Cu substrate, thereby reducing the extent of galvanic corrosion.^11−13^ As shown in Figure 5b, a capacity loss of 24 ± 1% was observed over 24 h for cells with 0.05 mAh/cm^2^, compared to an 8.1 ± 0.1% loss for cells with 0.1 mAh/cm^2^ capacity. Increasing the Li deposition further to 0.2 mAh/cm^2^ suppressed the capacity loss to just 5.2 ± 0.5%. These findings indicated that self-discharge at lower capacities, where Li coverage on the Cu substrate is sparse, is primarily driven by galvanic corrosion.

Comparing our measurements to nonaqueous liquid electrolyte-based studies highlights the potential differences in Li metal stability between liquid and dry solid-state polymer electrolytes. Zhou et al. reported approximately a 3.5% capacity loss after 5 h in Li metal cells containing 1 M LiFSI-DME electrolyte solutions cycled at a capacity of 0.1 mAh/cm^2^.^13^ In our study, cells with LiFSI-based SPEs cycled at the same capacity showed a 1.6 ± 0.5% loss after 3 h. It is important to note that SPE measurements are typically conducted at higher temperatures, which can accelerate corrosion rates. For instance, Lin et al. observed approximately 70–90% capacity loss over 48 h at 60 °C in cells containing 1 M LiTFSI DOL with 2% LiNO_3_ liquid electrolyte cycled at 0.05 mAh/cm^2^.^38^ Conversely, our LiFSI-based SPEs exhibited only a 45 ± 2% loss after 48 h, under the same temperature and capacity. These results emphasize the enhanced stability of Li metal deposits in SPE-based cells compared to those in liquid electrolyte systems.

**Figure 6:**
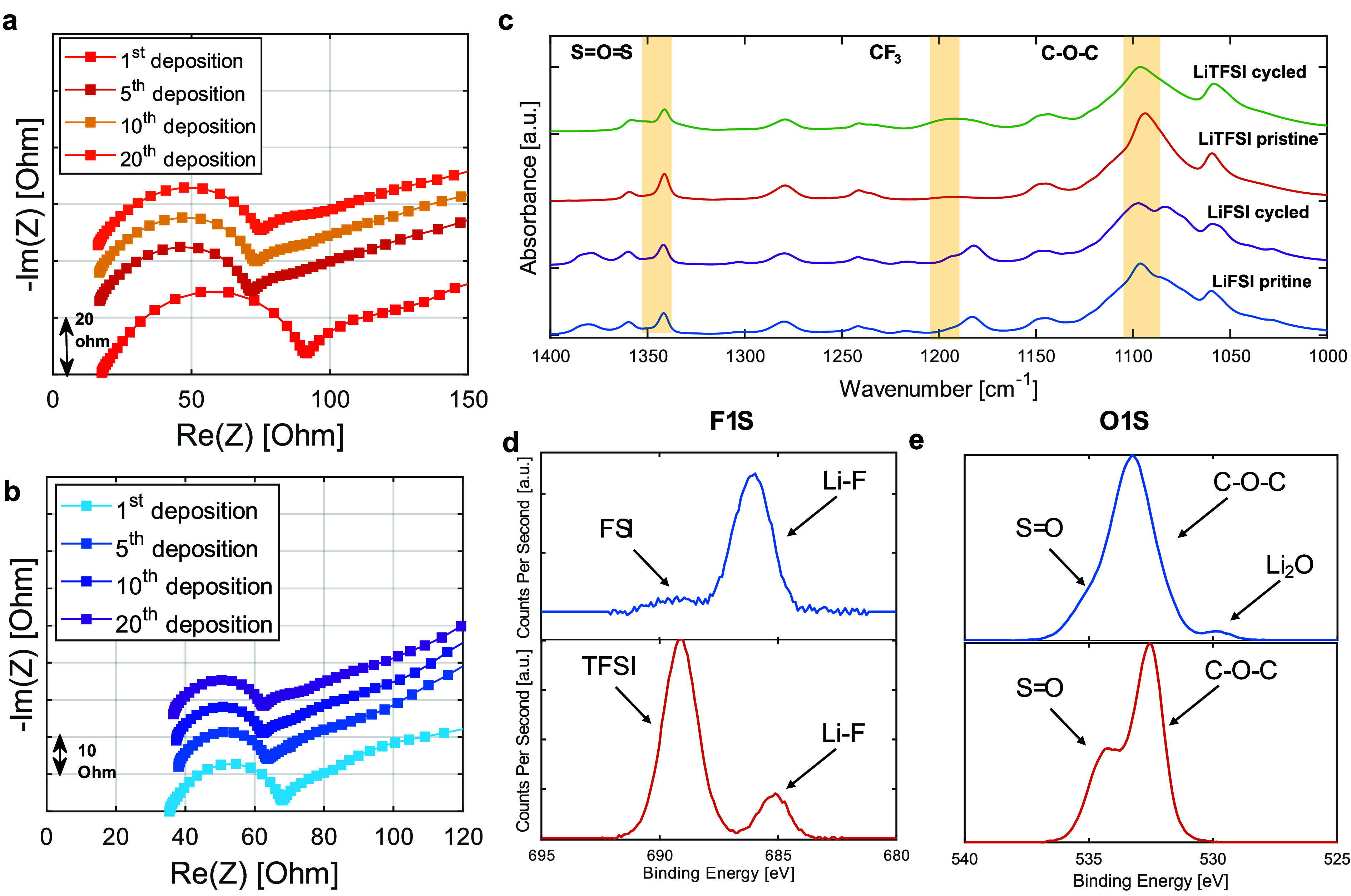
Nyquist plots from EIS measurements taken after Li-metal electrodeposition at various cycles for SPE-based cells with (a) LiFSI or (b) LiTFSI salts ([Li]/[EO] = 0.05), cycled at a current density of 0.1 mA/cm^2^ for 1 h at 60 °C. (c) FTIR spectra of Li-SPE interfaces after 20 deposition cycles ([Li]/[EO] = 0.05) at a current density of 0.1 mA/cm^2^ for 1 h. (d) F 1s and (e) O 1s XPS spectra from the Li-SPE interface following 20 deposition cycles for SPE-based cells with LiFSI or LiTFSI salts ([Li]/[EO] = 0.05) cycled at a current density of 0.1 mA/cm^2^ for 1 h.

To delve deeper into the underlying causes of the observed differences in CE, cycle lifetime, and deposit stability between the two Li salt-based SPEs, we utilized electrochemical impedance spectroscopy (EIS) which can show the evolution and dynamics of Li-SEPs interfaces.^40^ Our analysis concentrated on EIS responses from cells using both LiFSI and LiTFSI-based SPEs ([Li]/[EO] = 0.05). The Nyquist plots recorded after the first deposition across both SPE based cells reveal a semicircle spanning high to medium frequencies, transitioning into an incomplete, suppressed semicircle followed by a diffusion tail (Figure 6a and 6b). The semicircle’s starting point on the real axis at high frequencies is associated with bulk and uncompensated impedance, while the semicircle’s end point signifies the interfacial resistance (R_int_), representing charge transfer at the electrode–electrolyte interface.^41,42^ The appearance of additional semicircles at lower frequencies may represent heterogeneity in the interfacial resistance, suggesting a range of interfacial characteristics within the cell.^43^

In both SPE systems, the bulk and uncompensated impedance consistently remain stable throughout cycling, yet a distinct variation in the behavior of interfacial resistance is observed. Initially, the interfacial resistance is notably higher at the first deposition but decreases significantly in subsequent cycles. Despite the similar trends observed in both systems, the interfacial resistance values significantly differ. Averaging across several identical cells revealed that the initial interfacial resistance in the LiFSI system was approximately 40 ± 10 Ω, decreasing to 16 ± 7 Ω in the 20th cycle. Conversely, the LiTFSI-based system exhibited an initial resistance of 70 ± 20 Ω, which then lowered to 57 ± 3 Ω after 20 cycle. Moreover, after the initial deposition, the interfacial resistance in the LiFSI-based cell remained unchanged with further cycling, within the error bars, while in contrast to the LiTFSI-based cell resistance slightly increased with the cycle number from 54 ± 2 Ω after the fifth deposition to 57 ± 3 Ω after the 20th deposition.^44^ The EIS findings highlight a marked distinction in interfacial resistance stability between the two systems; the stability of the LiFSI system might be related to its longer lifetime compared to the LiTFSI system as the SEI remains stable over cycling. Additionally, the LiFSI system demonstrates a more favorable interphase for charge transfer, which likely accounts for the higher Coulombic efficiency observed in Li electrodeposition processes compared with LiTFSI-based cells.

To elucidate the chemical nature of the Li-SPE interphase formed during cell stabilization, we used ATR-FTIR to analyze the electrode surface after 20 electrodeposition cycles in both LiTFSI- and LiFSI-based SPE cells (Figure 6c). In LiTFSI-based SPE, we observed an increase in peaks associated with CF_3_ (1192 cm^–1^) and S=O=S (1350 cm^–1^), along with other signatures indicative of the formation of an interphase at the Li-polymer interface.^45^ The elevation in CF_3_ peaks could result from either an increased content of Li salt at the interface or partial decomposition of the TFSI anion. The peak at 1095.7 cm^–1^, corresponding to C–O–C, is influenced by the degree of coordination with alkali metal ions such as Li. Notably, we did not observe any significant shifts or formation of shoulder peaks, suggesting that the Li ions remain in a similar state and content as in the uncycled membrane. Furthermore, the emergence of a peak at 1350 cm^–1^, corresponding to the asymmetric stretch of S=O=S, indicates the potential breakdown of the LiTFSI salt into species such as Li-SO_2_CF_3_ and Li_2_-N-SO_2_CF_3_, as suggested by Nagasa et al.^46^

In the LiFSI samples, we observe vibrational features similar to those found in the LiTFSI samples, underscoring common chemical interactions within these systems. However, these membranes exhibited several unique spectral changes compared with the LiTFSI-based SPEs. These distinctive changes could imply discrepancies in the exact interface composition and interaction dynamics of each system. Specifically, a pronounced peak at 1083.8 cm^–1^ signals strong Li-PEO interactions within the amorphous phase, marking a significant difference in Li-polymer interactions.^47,48^ The decrease in the main peak at 1097 cm^–1^ and the redshifts and appearance of shoulders with cycling are in line with other reports on PEO based SPEs, suggesting that an amorphous Li^+^ containing layer promotes Li transport across the interphase. This coordination of Li with the PEO might also stabilize the PEO and contribute to a more anion derived SEI, which might be the cause of the increased stability observed in the EIS measurements. Furthermore, the pronounced shoulders at 954 cm^–1^ and 938 cm^–1^, are related to changes from the decrease in the O–C–O torsional angle necessary for the coordination of Li ions, which may indicate on alterations in the polymer backbone coordination at the Li-SPE interface.^48^ These alterations, indicative of increased Li ion content and PEO phase amorphization, might account for the lower impedance in LiFSI-based electrolytes compared with LiTFSI-based SPEs, as demonstrated in Figures 6a and 6b.

To gain a deeper understanding of the chemical nature of the Li-SPE interphase, we conducted X-ray photoelectron spectroscopy (XPS) measurements of the deposited Li metal after the cells had stabilized for 20 cycles (Figure 6d and 6e). In the F 1s spectra from the LiTFSI-based cells, we observed a peak centered around 689 eV, corresponding to fragmentation products related to fluorine-containing species, such as CF_3_ or SO_2_–CF_3_, or the original TFSI molecule within the formed interphase.^49−51^ Additionally, a relatively smaller broad peak centered at 686 eV is associated with Li–F bond from the presence LiF.^49,51^ In contrast, LiFSI-based cells exhibited a significantly stronger peak at 686 eV, with a smaller FSI-related peak centered around 688–689 eV. This suggests that in LiFSI-based cells, the salt predominantly reacts to form Li–F rather than other sulfonyl-containing fragments, which are more evident in LiTFSI-based cells.^49,52−54^ These results are consistent with previous reports on the enhanced formation of Li-F in LiFSI-containing electrolytes compared to LiTFSI.^55^ The O 1s spectra presented in Figure 6e show a prominent peak at 532.5 eV for both salts, corresponding to the C–O–C bond from the PEO backbone. In LiFSI, we observe an additional broad peak at 529.7 eV that can be associated with lithium oxide and alkoxides as part of the formed interphase.^54,56,57^ In contrast, for LiTFSI, we see a peak at higher binding energies, which can be associated with O=S=O (534.3 eV) from the sulfonyl-containing fragments of TFSI molecules.^54,58,59^

From the EIS, FTIR, and XPS results, we can now elucidate the detailed Li-SPE interphase composition and properties. Given the similar bulk transport properties of both SPEs, the observed performance differences are likely due to variations in the interphase between the SPE and the Li metal. The SEI in LiFSI cells shows more inorganic species, such as Li_2_O and Li–F, in good correlation with our FTIR results, which imply an increased presence of Li ions. The ionic conductivity of interphases coating these species is known to be high, as evidenced by the lower resistive interphase in the EIS. The presence of Li alkoxides is also known to form in Li-PEO interphases and increase the ionic conductivity of Li ions. The identification by FTIR and XPS spectroscopy of a sulfonyl-rich interphase in cells containing LiTFSI demonstrates that the breakdown of the anion is significantly different from that of FSI, despite their chemical similarity. The lack of a Li inorganic-rich interphase in the LiTFSI-based cells can explain the high resistivity of the interphase. Inefficient ionic transport can lead to uneven metal deposition, which may be the reason for the lower cycle life and Coulombic efficiency of reversible Li electrodeposition in LiTFSI-based cells.^60^ Furthermore, the lack of inorganic structural elements can explain the low stability of the interphase and the enhanced corrosion of the Li metal in LiTFSI cells compared with those of LiFSI-based cells.

The chemical investigation of the SEI composition and properties alongside the long-term cycling of the dry SPE cells provides valuable insights into the relationship between interphase formation and Li electrodeposition efficiency. These findings align with recent research on electrolyte solution-based cells. For example, Chen et al. proposed that an amorphous SEI enabling efficient ionic transport could stabilize Li electrodeposition, thereby enhancing efficiency.^61^ In addition, recent studies have suggested that LiTFSI-based cells are commonly believed to form an SEI that is comprised of more solvent-derived organic molecules, while LiFSI is believed to form a SEI richer in inorganic species, that are believed to improve interfacial conductivity.^23,62,63^ Our results corroborate findings from liquid electrolyte solution studies, confirming that subtle chemical changes at the Li-SPE interface, which form during Li electrodeposition, offer a viable strategy to enhance the performance of reversible Li electrodeposition in dry SPE-based cells.

## 3 Conclusions

This study presents a comprehensive evaluation of the efficiency, stability, and cycle lifetime of reversible Li electrodeposition in dry solid-state polymer electrolytes, aiming to benchmark performance metrics of Li|SPE|Cu cells and elucidate the governing physiochemical factors. Comparative analyses have underscored the correlation between increased Coulombic efficiency and higher current densities and salt concentrations. However, it was noted that lower values of these parameters were more conducive to extended cycle life, indicating a trade-off in simultaneously optimizing both metrics.

Key factors influencing these differences were identified, particularly the Li-SPE interphase, which mirrors the significant influence observed in liquid electrolyte solutions on the electrodeposition mechanism and efficiency. Our spectroscopic measurements (EIS, FTIR, and XPS) revealed that the SEI in LiFSI cells is lithium-rich, correlating with increased ionic conductivity and lower interfacial resistance. This is in contrast to the sulfonyl-rich interphase found in LiTFSI cells, which is associated with a higher resistivity and less stable SEI formation. Moreover, our corrosion studies demonstrated that the FSI-based interphase offers superior protection for the deposited Li metal, effectively suppressing the self-discharge processes during extended rest periods.

While our findings indicate significantly higher Li electrodeposition Coulombic efficiency compared with previous studies on dry SPEs, the efficiency still falls short of the benchmarks required for fully rechargeable batteries. Liquid and gel electrolyte studies have enhanced efficiency with additives; however, striving for a purely dry solid electrolyte system limits the use of such methods. Thus, further exploration of new Li salt combinations, concentrations, or molecular modifications to PEO-based electrolytes might provide a pathway to achieve the desired CE while preserving the solid-state integrity of the system.

## 4 Experimental and Methods

### Materials

Poly(ethylene oxide) (PEO, Mw 600 000 Da) and anhydrous Acetonitrile (ACN, ≥ 99.9%) were purchased from Sigma-Aldrich, and Cu foil (10 μm thickness) was purchased from Gelion PLC, cleaned by sonication in Ethanol and triple distilled water, then dried thoroughly. Li metal discs (16 mm⌀) were purchased from Gelion PLC. Lithium bis(fluorosulfonyl)imide (LiFSI) salt was purchased from Arkema. Lithium bis(trifluoromethane)sulfonimide (LiTFSI) was purchased from Gelion PLC. PEO was dried at 50 °C under vacuum overnight, Lithium salts were dried at 120 °C under vacuum overnight, and Acetonitrile was dried using molecular sieves (3 Å, Sigma-Aldrich).

### Solid Polymer Electrolyte Preparation

Pre-weighted amounts of PEO and Li salt were dissolved in ACN at 50 °C for 3 h under in Argon glovebox (Vigor, O_2_ was kept under 1 ppm and H_2_O under 0.05 ppm) by stirring separately, and then mixed and left to stir at 50 °C overnight to combine, subsequently the mixture was cast into PTFE molds and left to dry overnight, the membranes were transferred to vacuum oven (MeltPrep VChamber) to dry under vacuum at 50 °C overnight. The dry membranes were pressed at 90 °C and 100 bar for 1 min (Nug Smasher XP) in 100 μm thick moldes and punched to 19 mm⌀ discs.

### Electrochemical Measurements

Helf-cell configurations were assembled in stainless stell CR2032 coin-cells using a 12 mm⌀ Cu disc as a working electrode, a 19 mm⌀ SPE disc with a thickness of 100 μm, and 16 mm⌀ Li discs, and two stainless stell 1 mm thick spacers were added to maintain sufficient contact between the SPEs membranes and electrodes. All constant current cycling was done using a Neware CT-4008Tn-5 V50 mA-HW B battery testing cycler, in a climate chamber (60 °C) with 6h rest period before cycling and at a stripping cutoff voltage of 1 V and deposition cutoff voltage of -0.5 V. Average Coulombic efficiency was calculated as the average for the last 50 cycles before cell short circuit, for cells that did not stabilize enough cycles before short circuit only 25 cycles were considered, average efficiency and cycle lifetime were calculated over a minimum of three cells for every set of parameters.

Electrochemical impedance measurements were carried out inside the constant climate chamber using a VSP-3e Biologic potentiostat at an amplitude of 100 mV, recording 8 points per decade. The equivalent circuit consists of two parallel circuits in series to control the ohmic resistance of the connections. The first parallel circuits are a resistor in parallel with a constant phase element, which represent the charge-transfer processes; these circuits are connected in series to a Warburg impedance consistent with diffusion. Transference number was measured according to the method suggested by Bruce et al.^64^ using a 10 mV polarization amplitude, the measurements were conducted using a VSP-3e Biologic potentiostat at 60 °C. Corrosion measurements were carried out using the Neware battery testing cycler, the cells were first cycled for 30 cycles at 0.1 mA/cm^2^ for the appropriate capacity at 60 °C, followed by a rest period that was applied for a set interval, the calls were then cycled for 10 more cycles for stabilization followed by longer rest cycles repeatedly. Fitting was done to a first order kinetic exponential decay, with R^2^ > 0.99. The ionic conductivity of the SPEs was extracted from EIS measurements performed using a Gamry 600+ reference potentiostat, with the SPE films placed on gold blocking interdigitated electrodes (IDE) inside an argon-filled glovebox, as described by the work of Sharon et al.^65^

### Spectroscopic Measurements

Coin cells were opened using the Tob electric crimper, and Cu substrate after Li electrodeposition was gently peeled off the SPE and measured by a Thermo scientific Nicolet iS50 FT-IR with ATR accessory. XPS spectra of Li and residuals on Cu was gathered using an X-ray Photoelectron Spectroscope Axis Supra (Kratos) under an argon environment, and samples were transferred to the measurement instrument using a sealed transfer cell.

### SEM Imaging

Cells were cycled at the mentioned time and current as specified for each image. Imaging of the Cu substrate after electrodeposition was done using an Analytical High Resolution Scanning Electron Microscope Apreo 2S (Thermo Fisher Scientific) at a 0.1 nA and 5 kV acceleration voltage.

## Supporting Information Available

Pressing SPE advantages, conductivity measurements, voltage profiles of different current and concentration, transference number measurements, detailed corrosion measurements, picture of SPE, full FTIR spectra (PDF)
