# Supplementary material for: Enhancing Efficiency, Stability, and Cycle Life of Lithium Metal Electrodeposition in Dry Solid-State Polymer Electrolytes
Source: ACS Appl Mater Interfaces. 2024 Nov 20;16(48):66159–69. doi: 10.1021/acsami.4c15287 (PMC11622184; doi:10.1021/acsami.4c15287)
Supplement: Supplementary file 1 — am4c15287_si_001.pdf [file am4c15287_si_001.pdf]

Supporting information

Enhancing Efficiency, Stability, and Cycle Life of Lithium Metal Electrodeposition in Dry Solid-State Polymer Electrolytes

Idan Bar-lev[1], Keren Shwartsman[1], Vivek Kumar Singh[1], Netta Bruchiel-Spanier[1], Emily Ryan[2], Netanel Shpigel[3] and Daniel Sharon*[1].

Corresponding author: Daniel.sharon@mail.huji.ac.il

1. Institute of Chemistry, The Hebrew University of Jerusalem, 9190401, Jerusalem, Israel
2. Department of Mechanical Engineering; Division of Materials Science and Engineering; Institute for Global Sustainability, Boston University, Boston, MA 02215
3. Department of Chemical Sciences, Ariel University, Ariel 40700, Israel.

Additional Results and Figures

Our investigation into the CE and cycle life of SPEs fabricated via solvent cast pressed and unpressed membranes, with a Li:EO ratio of r=0.05—revealed that the fabrication technique exerts minimal impact on CE, which stabilized at 78.1±0.4% at 0.05 mA/cm² (**Figure S1**). However, both hot-pressed and dry-pressed SPEs showcased a marked improvement in cycle life, averaging at 180±20 cycles compared to 50±5 cycles for non-pressed membranes. Interestingly, no significant performance disparity was observed between hot-pressed and dry-pressed membranes. As a result, we opted to hot press all SPEs discussed in this manuscript, highlighting their beneficial impact on the cells' overall performance and longevity.

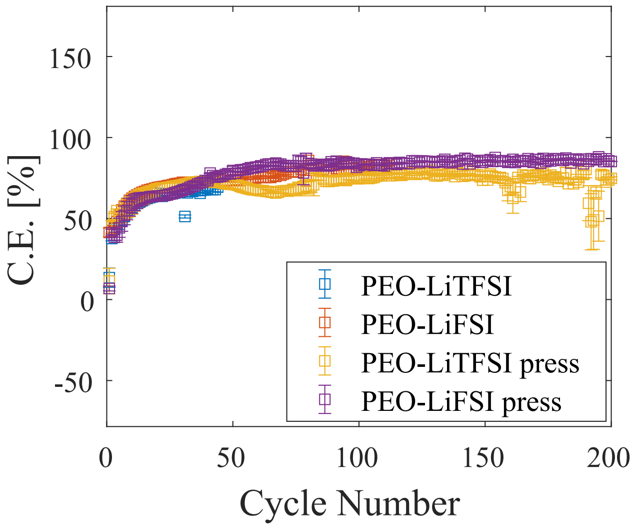

Figure S1. Coulombic efficiency as a function of cycle number for different SPE preparation methods in Li|SPE|Cu cells with a salt concentration of r = 0.05, cycled at a current density of 0.05 mA/cm² and 60 °C.

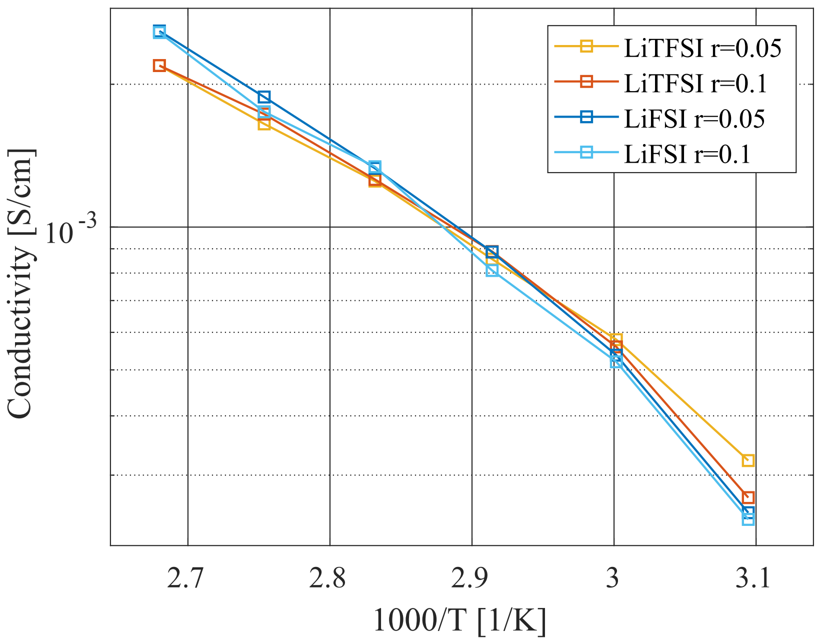

Figure S2. Ionic conductivity of different SPEs as function of temperature.

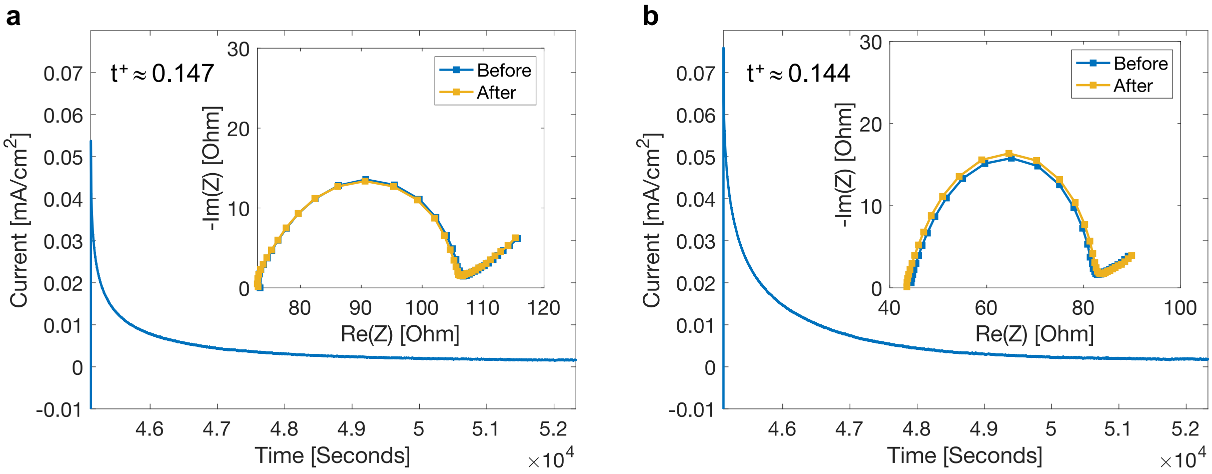

Figure S3. Transference number measurements, conducted by measuring the impedance spectra before and after polarization, for (a) LiTFSI and (b) LiFSI membranes.

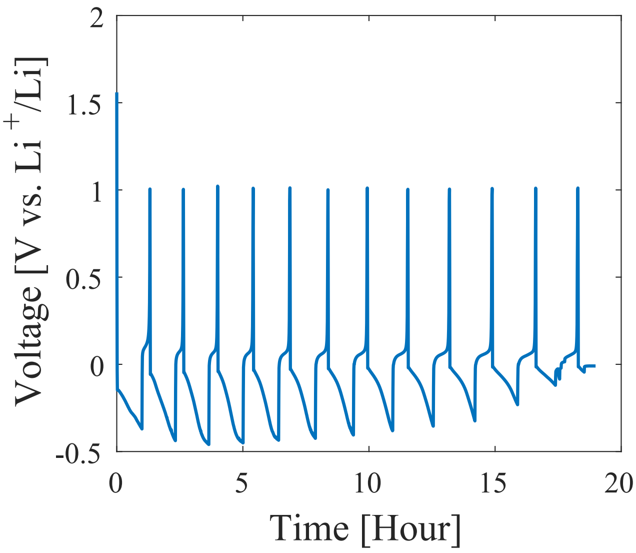

Figure S4. Voltage profile of Li|PEO-LiFSI|Cu cell at r=0.05 cycled at 0.15 mA/cm^2 at 60 °C.

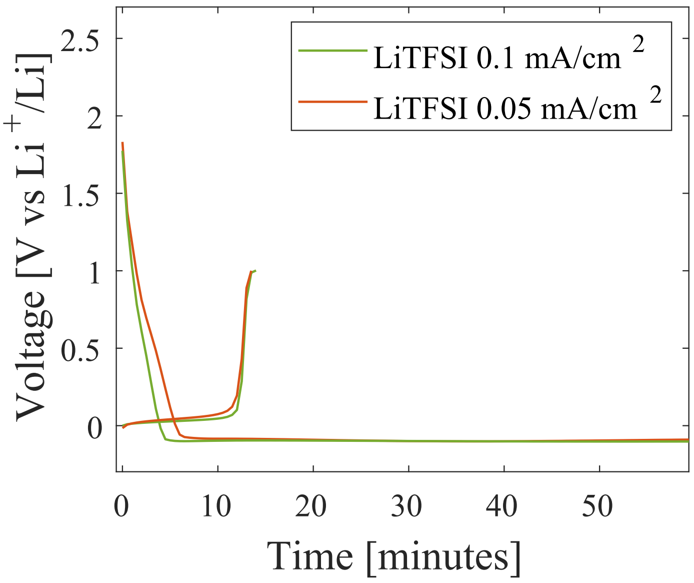

Figure S5. First deposition and stripping voltage profile of Li|PEO-LiTFSI|Cu (r=0.05) cells at 60 °C.

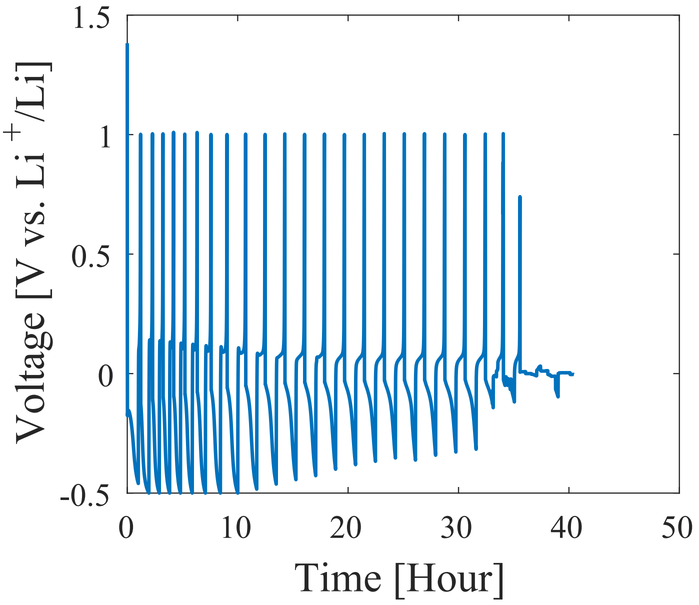

Figure S6. Voltage profile of Li|PEO-LiFSI|Cu cell at r = 0.16 ratio cycled at 0.1mA/cm^2 at 60 °C.

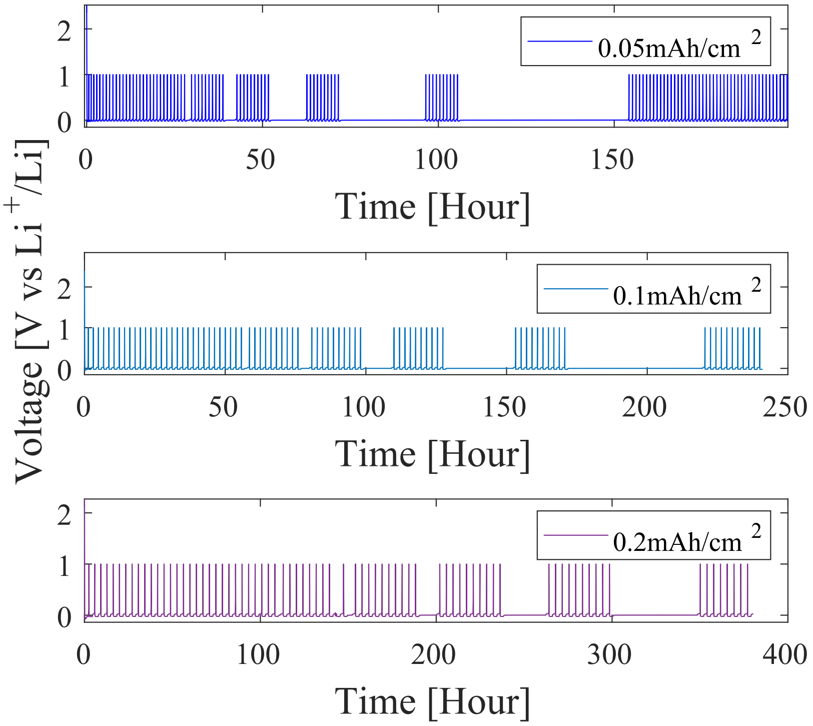

Figure S7. Voltage profiles of self-discharge protocol for LiFSI based cells cycled at current density of 0.1mA/cm^2 at varying capacities as indicated in the figure, at 60 °C.

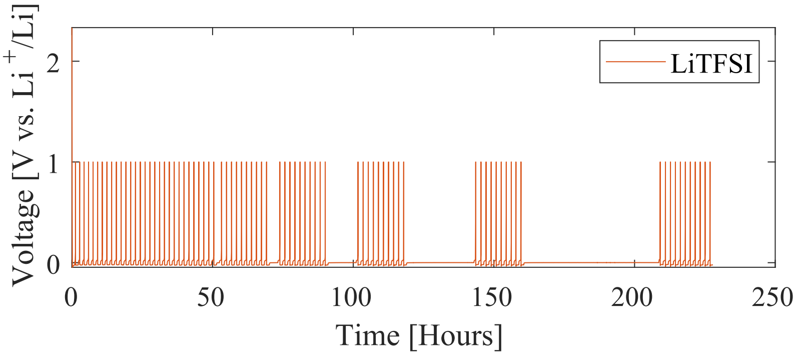

Figure S8. Self-discharge protocol of PEO- LiTFSI based cells cycled at current density 0.1mA/cm^2 at 60 °C.

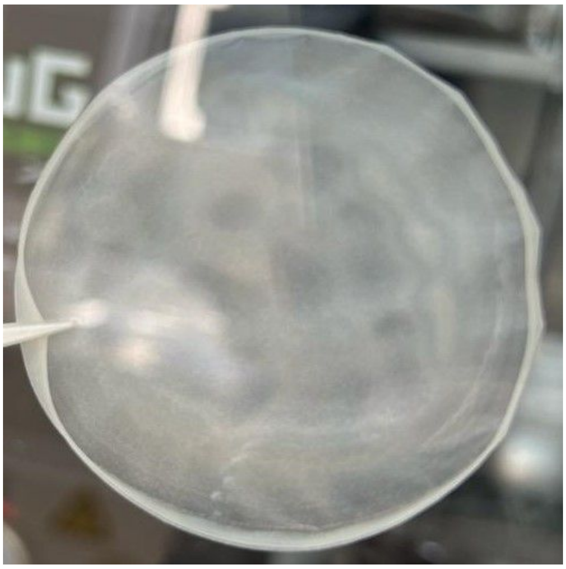

Figure S9. Photo of SPE membrane.

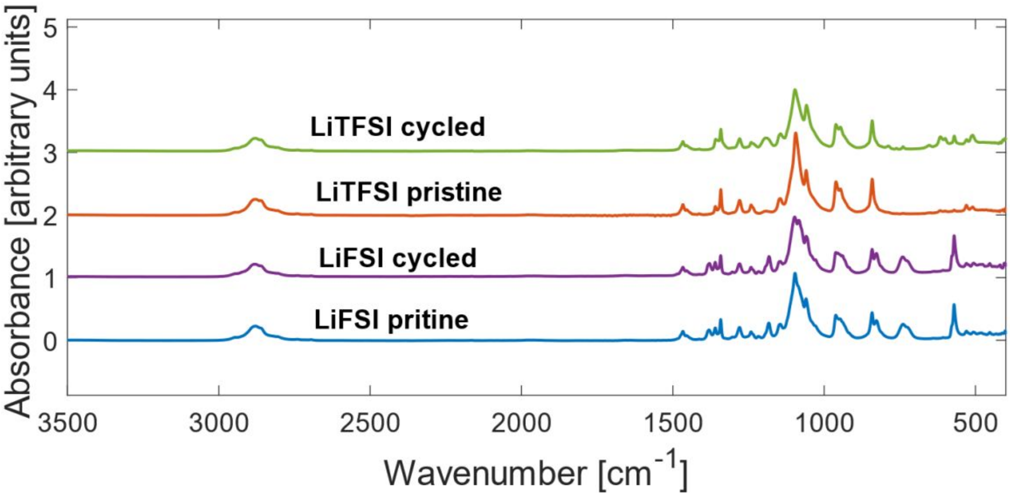

Figure S10. Full FTIR spectra of pristine Li salts and cycled SPEs samples.
